# Effect of Moxidectin on Bed Bug Feeding, Development, Fecundity, and Survivorship

**DOI:** 10.3390/insects8040106

**Published:** 2017-09-30

**Authors:** Chen Zha, Changlu Wang, Johnathan Michael Sheele

**Affiliations:** 1 Department of Entomology, Rutgers-The State University of New Jersey, New Brunswick, NJ 08901, USA; cz166@scarletmail.Rutgers.edu; 2 Department of Emergency Medicine, University Hospitals Cleveland Medical Center & Case Western Reserve University, Cleveland, OH 44106, USA; jsheele@gmail.com

**Keywords:** *Cimex lectularius*, moxidectin, pest management

## Abstract

The common bed bug, *Cimex lectularius* L. (Hemiptera: Cimicidae), is a blood-feeding ectoparasite which experienced world-wide resurgence during recent decades. The control of bed bugs is often challenging, due to their cryptic nature and resistance to commonly used insecticides. In this study, we evaluated the effect of the antiparasitic drug moxidectin on bed bug survival, reproduction, and development. The LC_50_ (lethal concentration to kill half the members of a tested population) of moxidectin against bed bug male adults, female adults, and large nymphs were 52.7 (95% CI (confidence interval): 39.5–70.8), 29.3 (95% CI: 20.7–40.5), and 29.1 ng/mL (95% CI: 23.3–35.3), respectively. Moxidectin (≥ 25 ng/mL) reduced egg laying of bed bug females, but showed no significant effect on egg hatching. One time feeding on rabbit blood containing 20 and 40 ng/mL moxidectin showed no negative effects in bed bug feeding and blood meal ingestion, but significantly reduced digestion rates and nymph molting rates. Although moxidectin at concentrations of 20 and 40 ng/mL only caused moderate mortality in bed bugs, it significantly interrupted digestion, development, and oviposition of survived bed bugs for at least one week after feeding. Moxidectin is a promising supplement of the existing bed bug control materials if its use on humans can be approved in the future.

## 1. Introduction

The common bed bug, *Cimex lectularius* L. (Hemiptera: Cimicidae), is a blood-feeding ectoparasite which experienced world-wide resurgence during recent decades [1,2,3,4]. Bed bug bites cause itchiness, rashes, anxiety, sleeplessness, and other psychological sequelae [5,6]; people who had little or no reaction to bed bug bites can be sensitized after repeated exposure [7]. Public tolerance of bed bugs is almost zero, and social stigma and economic hardship are often associated with bed bug infestations [8]. Control of bed bugs is often challenging, one reason is that they are good at hiding, therefore very thorough inspection and treatments are required for successful management [8]. Another factor is that resistance to insecticides has been widely established among bed bug field populations. Pyrethroids are the mainstay chemicals used for bed bug control over the past decades [4]. However, the resistance to pyrethroids has become widespread in bed bug populations across the United States [3,4,9]. In spite of the many bed bug control methods and materials that have been developed [10,11,12], new and effective treatments are needed to improve the efficacy of the current bed bug management programs.

Instead of exposing bed bugs to insecticides by contact, delivering chemical agent via oral ingestion has been explored as a potential bed bug management strategy. Sierras and Schal (2017) [13] treated bed bugs by feeding them blood containing abamectin, clothianidin, fipronil, and indoxacarb; all the tested insecticides except indoxacarb caused rapid mortality in bed bugs, and fipronil was ∼ 43-fold more effective by ingestion than by topical application. The results showed the potential of developing ingestible liquid baits for bed bug management. However, the technique of developing bed bug liquid bait is not available yet, not to mention the possible cost and efficiency. Bed bugs are ectoparasites so an oral human antiparasitic drug potentially holds promise for insect control.

The potential of oral human antiphrastic drugs to kill bed bugs was studied recently [14]. Sheele and Ridge (2016) [15] fed bed bugs with blood containing ivermectin and moxidectin, and found that blood treated by higher concentrations of >25 ng/mL ivermectin or moxidectin caused significantly higher mortality (50–100%) in bed bugs than the controls (0–6%) by day 13. Besides direct mortality, bed bugs treated by ivermectin and moxidectin also showed signs of sub-lethal effects including reduced fecundity, feeding difficulty, and incomplete ecdysis.

Moxidectin is derived from nemadectin, an active fermentation milbemycin product isolated from *Streptomyces cyanogriseus* in 1983 [16]. It has been widely used in cattle, sheep, and companion animals for parasite treatments [17,18]. It is effective against a wide range of parasites including cyathostomes and other equine parasites [19], *Muellerius capillaris* (hair lungworm) [20], ascarids, strongyles, tapeworms [21], sarcoptic, demodectic, psoroptic mites [18], as well as human lymphatic filarial worms [22]. Based on previous studies with nematodes, the primary mechanism of moxidectin is binding to invertebrate-specific glutamate-gated chloride channels, leading to paralysis and death of the parasite due to hyperpolarization of nerves and muscle fibers [23,24]. Although not yet U.S. Food and Drug Administration approved for human use, moxidectin is safe and well tolerated in humans in a single oral dose between 3–36 mg [25]. It is currently being studied for treating onchocerciasis (river blindness) and scabies, with positive results in both safety and efficacy [26,27].

The pharmacokinetics of oral moxidectin in human plasma has been studied in detail by Cotreau et al. 2003 [25]. For doses between 3–36 mg, the time of maximum concentration varied from 1.8–4 h, with a long plasma half-life (20–35 days). After a single oral dose of 36 mg, the mean concentration of moxidectin in plasma was around 40 ng/mL at 2 days, 10 ng/mL at 8 days, and then dropped to 1–10 ng/mL during the following 8–80 days. Unlike endoparasites, bed bugs will only be exposed to moxidectin when the insect feeds on a person that has taken the drug, which may be hours or days after drug ingestion. Therefore, the long plasma half-life may make moxidectin a promising drug against bed bugs.

Although the effect of moxidectin on bed bugs was reported in a preliminary study [15], information on the toxicity and sub-lethal effects of moxidectin is still very limited due to the small sample size used. In this study, we fed bed bugs with rabbit blood containing different concentrations of moxidectin, and then evaluated its effect on bed bug feeding, development, fecundity, and survivorship. We determined the LC_50_ (lethal concentration to kill half the members of a tested population) of moxidectin against different sexes and stages of bed bugs, and measured the impacts of moxidectin on bed bug feeding, ingestion, and digestion. We also determined the effects of moxidectin on female oviposition, as well as egg and nymph development.

## 2. Materials and Methods

### 2.1. Chemicals

Cydectin ^®^ Injectable Moxidectin (Boehringer Ingelheim Vetmedica Inc., Duluth, GA) containing propylene glycol (50–75%), ethanol (20%), and 1% moxidectin was used in moxidectin treatment groups. Hanks’ Balanced Salt Solution (HBSS) without calcium, magnesium, or phenol red (Corning Cellgro, Manassas, VA) was used as buffer solution for dissolving the 1% moxidectin stock solution before mixing with blood.

### 2.2. Insects

A field strain of *C. lectularius* (Irvington strain) was used in this study. It was collected from multiple apartments during 2012–2013 in a building in New Jersey, and was moderately resistant to pyrethroid insecticides in our preliminary laboratory assay in January 2016. Bed bugs were maintained in plastic containers (5 cm diameter and 4.7 cm height; Consolidated plastics, Stow, OH) with folded construction paper (40 mm length and 30 mm width) as harborages and held at 26 ± 1 °C, 40 ± 10% relative humidity (RH), and a 12:12 h (light:dark) photoperiod. They were fed biweekly on defibrinated rabbit blood (Hemostat Laboratories, Dixon, CA) using a Hemotek membrane-feeding system (Discovery Workshops, Accrington, UK). Rearing containers were modified into feeding jars by cutting the bottom off and attaching a fine nylon mesh, through which bed bugs can feed on the blood source. Bed bugs were fed one week prior to all experiments.

### 2.3. Experiment 1: Effect of Moxidectin on Bed Bug Morality, Female Fecundity, and Egg Development

Engorged bed bug males, females, and nymphs were selected after feeding, they were then used in the experiment one week later. Medium to large nymphs (third to fourth instars) were selected. Different sexes and stages were contained separately in individual feeding jars. The numbers of males, females, and nymphs included in each trial were 15, 15, and 30, respectively. During experiment, bed bugs were fed with blood containing the following concentrations of moxidectin: 2.5, 5, 25, 50, 100, and 150 ng/mL. Moxidectin stock solution (1%) was diluted in HBSS buffer solution before mixing with blood; the final mixture contained 99.96% blood and 0.04% HBSS and moxidectin by volume. Control groups were fed with blood containing the same volume of HBSS only. Each treatment was replicated three times.

Bed bugs were fed for 30 min; the numbers of engorged bed bugs were recorded, and unfed bed bugs were excluded from the study. Females were moved to new harborages for oviposition observation. After treatment, bed bugs were kept in an incubator (Percival Scientific Inc., Perry, IA) which maintained 26 ± 1 °C, 40 ± 10% relative humidity (RH), and a 12:12 h L:D photoperiod. Mortalities were recorded after one week, and eggs laid by females during the seven day period were observed for 10 d for hatching rates.

### 2.4. Experiment 2: Effect of Moxidectin on Bed Bug Feeding, Digestion, and Nymph Development

Bed bugs were selected and prepared as in Experiment 1 and fed with rabbit blood containing 20 and 40 ng/mL moxidectin. The two concentrations were chosen to represent moderate to high moxidectin levels in human blood plasma after a single 36-mg oral dose within the first week [25]. Control groups were fed with rabbit blood containing the same volume of HBSS only. Treated rabbit blood and bed bugs were prepared following the same protocol as in Experiment 1. The numbers of males, females, and nymphs included in each trial were 15, 15, and 30, respectively. Each treatment was replicated three times.

Bed bugs were fed for 30 min, the numbers of engorged bed bugs were recorded, and unfed bed bugs were excluded from the study. Bed bugs were kept in an incubator after treatment as in Experiment 1. Bed bug males and females were weighed on a balance (Mettler-Toledo Inc., Washington Crossing, PA) in groups of 15 before feeding. After feeding, only engorged bed bugs were weighed. The moralities were recorded after one and two weeks, and live bed bugs were weighed again in groups.

Nymphs treated in Experiment 2 were observed for molting process at one and two weeks after feeding. The numbers of exuviae and live nymphs were recorded to evaluate the molting rate. The feeding and digestion rates of nymphs were not evaluated because the size of the nymphs varied.

### 2.5. Experiment 3: Repeated feeding on Moxidectin-Treated Blood

Survived bed bugs in Experiment 2 were fed with rabbit blood containing the same moxidectin concentration again at two weeks after the initial treatment, and the experimental protocols in Experiment 2 were repeated. The experiment stopped after second feeding because there were not enough survived bed bugs left.

### 2.6. Data Analysis

Probit analysis was used to calculate LC_50_ values of moxidectin against bed bug males, females, and nymphs. Since the lower concentrations (2.5–50 ng/mL) and higher concentrations (100 and 150 ng/mL) were tested separately with controls, all mortalities were corrected by Abbott’s formula before Probit analysis. The numbers of eggs laid per survived female and egg hatching rates were compared by Analysis of Variance (ANOVA) followed by Tukey’s HSD (honest significant difference) test.

The effect of moxidectin on bed bug feeding and digestion was evaluated by calculating feeding rate, ingestion rate, and digestion rate:

(1) $$Feeding\;rate=\frac{number\;of\;engorged\;bed\;bugs\;after\;feeding}{number\;of\;bed\;bugs\;before\;feeding}\times 100\%$$

(2) $$Ingestion\;rate=\frac{average\;weight\;gain\;after\;feeding}{average\;body\;weight\;before\;feeding}\times 100\%$$

(3) $$Digestion\;rate=\frac{average\;weight\;after\;feeding-average\;weight\;after\;digesting\;period}{average\;weight\;gain\;after\;feeding}\times 100\%$$

Feeding rates, ingestion rates, and digestion rates of bed bugs fed on 0, 20, and 40 ng/mL moxidectin were analyzed by ANOVA followed by Tukey’s HSD test. In Experiment 2, the digestion rate of females fed on 40 ng/mL moxidectin was not analyzed because the number of survived bed bugs in some groups was too small (<5). In that case, bed bugs fed on 0 and 20 ng/mL moxidectin were compared by Student’s *t*-test.

The effect of moxidectin on bed bug development was evaluated by calculating nymph molting rate:

(4) $$Nymph\;molting\;rate=\frac{number\;of\;exuviae}{number\;of\;survived\;nymphs}\times 100\%$$

The effect of moxidectin treatments on molting rates of bed bug nymphs was compared by ANOVA followed by Tukey’s HSD test. The corrected mortalities of bed bug males, females, and nymphs were compared by two-way ANOVA followed by Tukey’s HSD test. All data met the assumption of normal distribution and therefore no transformation was needed.

Bed bug mortalities and molting rates in Experiment 3 were summarized but not analyzed for statistical differences because the numbers of survived and fed again in many treatment groups were too small. All analyses were conducted using SAS software 9.3 [28].

## 3. Results

### 3.1. Toxicity of Moxidectin Against Bed Bugs

The LC_50_ values of moxidectin against bed bug males, females, and large nymphs were 52.7 (95% CI: 39.5-70.8), 29.3 (95% CI: 20.7–40.5), and 29.1 (95% CI: 23.3–35.3) ng/mL, respectively (Table 1). The LC_50_ value for nymphs was significantly lower than that for males. The 95% CI of LC_50_ value for females slightly overlapped with that for males, but there was a trend that females had a lower LC_50_ value.

### 3.2. Sub-Lethal Effects of Moxidectin on Bed Bugs

Starting from 5 ng/mL, moxidectin treatment significantly reduced fecundity of survived females (Table 2). At 50 ng/mL concentration, moxidectin caused sterility of survived females. Moxidectin had no detectable effect on the survival of eggs.

Moxidectin at 20 and 40 ng/mL did not significantly affect the feeding rate of bed bug males, females, and nymphs (Table 3). They significantly increased the ingestion rate of males. The digestion rates of both males and females were significantly reduced by moxidectin treatment, with females affected at a lower concentration than males.

There were 86, 58, and 39 nymphs survived at two weeks after feeding on blood containing 0, 20, and 40 ng/mL moxidectin, respectively. The nymph molting rates at one week after feeding on blood containing 0, 20, and 40 ng/mL moxidectin were 99 ± 1, 3 ± 2, and 0 ± 0%, respectively. Both 20 and 40 ng/mL moxidectin treatments caused significant lower molting rate compared with that in the control (F = 2067.22; df = 2, 6; *p* < 0.001; Tukey’s HSD test, *p* < 0.05). The nymph molting rates at two weeks after feeding on blood containing 0, 20, and 40 ng/mL moxidectin were 100 ± 0, 84 ± 8, and 48 ± 16%, respectively. The molting rate in the 20 ng/mL moxidectin treatment became similar to that in the control, whereas the molting rate in the 40 nm/mL treatment remained significantly lower than that in the control (F = 6.87; df = 2, 6; *p* = 0.028; Tukey’s HSD test, *p* < 0.05).

### 3.3. Differential Susceptibility of Males, Females, and Nymphs to Moxidectin

At 1 week after treatment, the mortalities of bed bug males, females, and nymphs in control groups were 5 ± 2, 2 ± 2, and 0 ± 0%, respectively. The mortalities of bed bug males, females, and nymphs fed on 20 ng/mL moxidectin were 7 ± 5, 50 ± 9, and 30 ± 4%, respectively. The mortalities of bed bug males, females, and nymphs fed on 40 ng/mL moxidectin were 31 ± 7, 82 ± 5, and 47 ± 3%, respectively.

The mortalities of bed bugs in different treatments were significantly different (F = 97.48; df = 2, 18; *p* < 0.001). There was also a significant difference among bed bugs in different sexes and stages (F = 29.80; df = 2, 18; *p* < 0.001). There was a significant interaction between treatments and bed bug sexes/stages (F = 8.8; df = 4, 18; *p* < 0.001). The mortality of females that were fed with 20 ng/mL moxidectin was significantly higher than males; the mortality of females that were fed with 40 ng/mL moxidectin was significantly higher than males and nymphs (Tukey’s HSD test, *p* < 0.05).

### 3.4. Morphological Changes of Bed Bugs after Moxidectin Ingestion

**Figure 1:**
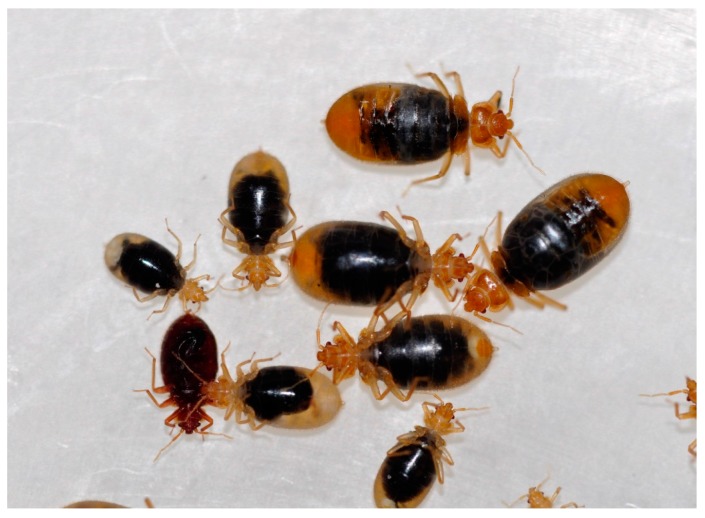
Bed bugs died after ingesting moxidectin-treated rabbit blood. Picture was taken seven day after treatment.

Intoxicated bed bugs became inactive and paralyzed at first, and subsequently died in relaxed positions (Figure 1). Their abdomen showed separation of clear liquid and black matter after death, possibly due to coagulation of indigestible blood in the gut.

**Figure 2:**
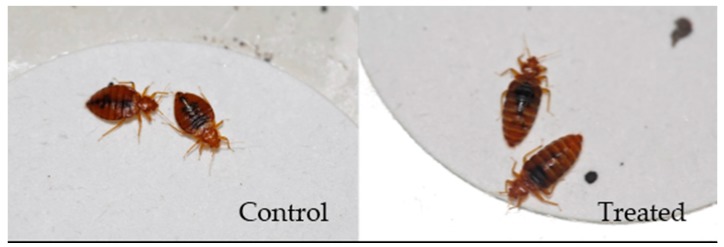
Bed bug males survived at one week after ingesting untreated rabbit blood and rabbit blood containing 40 ng/mL moxidectin.

Bed bugs survived at one week after ingesting control blood and blood containing 40 ng/mL moxidectin are shown in Figure 2. Compared to control groups, bed bugs fed with moxidectin-treated blood were still engorged with blood, indicating suppressed digesting process.

### 3.5. Effect of Repeated Moxidectin Treatment

During the second treatment, the feeding rates of bed bug males fed on control, 20, and 40 ng/mL moxidectin were 95 ± 3, 75 ± 18, and 43 ± 17%, respectively. There was no significant difference among the treatments (F = 3.59; df = 2, 6; *p* = 0.095). The feeding rates of bed bug nymphs fed on control, 20, and 40 ng/mL moxidectin were 37 ± 8, 55 ± 5, and 17 ± 17%, respectively. There was no significant difference among the treatments (F = 2.99; df = 2, 6; *p* = 0.126). The feeding rates of females were not analyzed due to low survivorship. The mortalities of males, females, and nymphs after the second moxidectin treatment are shown in Table 4. Although the small sample sizes did not allow for statistical analysis, the data indicate low mortality occurred from the second moxidectin treatment. 

The nymph molting rates after the second moxidectin treatment are shown in Table 5. The treatment apparently delayed the molting of nymphs, but the final molting rates after two weeks was similar.

## 4. Discussion

Moxidectin showed insecticidal activity against bed bugs when ingested through a blood meal. Its toxicity against bed bug females and nymphs was higher than that for adult males. Besides immediate insecticidal activity, moxidectin reduced the fecundity of bed bug females. Moxidectin treatment also decreased the fecundity of the survived females. No oviposition occurred during one week after feeding when the concentration of moxidectin reached 50 ng/mL. On the other hand, the hatching rates were not significantly affected by moxidectin.

No feeding deterrence of moxidectin was found at lethal concentrations. In fact, the males in treated groups ingested more blood meal than the control group, while there was no significant difference among females that received different treatments. The lower ingestion rate among males in the control was possibly due to experimental errors. Additional experiments would be required to determine the effect of moxidectin on feeding rates in bed bug males.

Although moxidectin did not show any negative effects in bed bug feeding and blood meal ingestion, bed bugs that ingested moxidectin suffered from decreased digestion. Previous studies show that the mode of action of moxidectin in killing nematodes is binding to invertebrate-specific glutamate-gated chloride channels, leading to paralysis and death due to hyperpolarization of nerves and muscle fibers [23,24]. Our observation in bed bugs was congruent with that conclusion: treated bed bugs became paralyzed and died slowly with blood meals un-digested; survived bugs could not digest, molt, or lay eggs probably due to suppressed muscle and neuro activities. At one week after feeding on treated blood, survived bed bug males, females, and nymphs were still engorged, while bed bugs fed on untreated blood had already digested the blood meals with abdomens flattened, indicating a result of interrupted digestion. Further studies using molecular biology methods are needed to determine if the mode of action of moxidectin in bed bugs is the same as in nematodes.

Moxidectin also slowed down the development of nymphs. At one week after treatment, the molting rates of bed bug nymphs in 20 and 40 ng/mL moxidectin treatments were significantly lower than the control. At two weeks after treatment, the molting rate of bed bug nymphs in the 40 ng/mL moxidectin treatment was still significantly lower than control, while the molting rate in the 20 ng/mL moxidectin treatment increased to the same level as control, indicating that bed bug nymphs fed on 20 ng/mL moxidectin could recover after two weeks.

At the second feeding, the feeding rates of males and nymphs in control and treatments were not significantly different; the relatively low feeding rates in nymphs were likely caused by environment factors since the control feeding rate was low as well. The mortalities of re-treated bed bugs were low, and most treated nymphs were able to molt after two weeks. The results indicate that after repeated moxidectin treatment, some bed bugs will still be able to survive and recover. Further feeding experiments at a larger scale are needed to determine the long-term sub-lethal effects and potential resistance development of bed bugs against moxidectin.

In our experiment, 40 ng/mL moxidectin only caused 31 ± 7, 82 ± 5, and 47 ± 3% mortalities in males, females, and nymphs, respectively, and the concentration of moxidectin in human plasma drops below 40 ng/mL two days after oral ingestion [25]. Therefore, it is unlikely that bed bugs can be eliminated by taking moxidectin alone. On the other hand, these in vitro feeding experiments cannot determine if any of the moxidectin metabolites have antiparasitic activity (or activity against bed bugs) as has been suggested for ivermectin [29]. Potential drug-drug interactions and drug-food interactions in vivo may exist for moxidectin as well. If the pharmacodynamic effects of moxidectin exceed the pharmacokinetics then we may expect moxidectin to cause higher bed bug toxicity with clinical use. Furthermore, the doses of moxidectin used in our experiments were based on the known pharmacokinetics of the drug in human plasma, but there may be differences in the concentration of moxidectin in the plasma compared to whole blood [30]. This highlights a limitation of extrapolating our expected bed bug mortality from in vitro feeding experiments to what could be seen in vivo. Nevertheless, moxidectin at concentrations of 20–40 ng/mL not only caused direct mortality to bed bugs, but also significantly suppressed the digestion, development, and oviposition of survived bed bugs for at least one week after feeding. Unlike most other bed bug control methods, taking moxidectin requires little, if any, effort for residents. Because bed bugs will keep engorged for longer periods after moxidectin ingestion, they may be more susceptible to other treatments such as insecticide application or steam application. Future studies on the interactions between moxidectin treatment and other treatment methods would help us to better understand the potential of using moxidectin in bed bug management.

## 5. Conclusions

Moxidectin not only causes direct morality in bed bugs, but also reduces the fitness of bed bugs by causing various sub-lethal effects, including reduced digestion, development, and fecundity. A small portion of bed bugs may be able to survive a blood meal with moxidectin at a concentration of 20–40 ng/mL. Therefore, moxidectin may not be a standalone effective bed bug control method, but shows potential as a supplementary method in bed bug management. If moxidectin can be approved for use in humans in the future, there is a possibility that bed bugs could be added to the list of target parasites.

## Figures and Tables

**Table 1:** Probit analysis results of toxicity of moxidectin against bed bugs.

Sex/Stage	*N*	Slope ± Standard Error	LC_50_ (95% Confidence Interval) (ng/mL)	χ^2^	*p*
**Male adult**	230	2.70 ± 0.38	52.7 (39.5−70.8)	50.4	<0.001
**Female adult**	214	2.46 ± 0.32	29.3 (20.7−40.5)	58.6	<0.001
**Nymph**	332	3.93 ± 0.42	29.1 (23.3−35.3)	88.4	<0.001

**Table 2:** Effect of moxidectin on bed bug female fecundity and egg hatching after 1 week.

Moxidectin Treatment (ng/mL)	Control	2.5 ng/mL	5 ng/mL	25 ng/mL	50 ng/mL	ANOVA Statistics
**No. of survived females**	30	35	31	13	14	-
**Eggs laid per survived female**	11.0 ± 0.7a †	10.5 ± 0.4a	7.6 ± 0.9b	0.6 ± 0.1c	0 ± 0c	F = 101.22; df = 4, 10; *p* < 0.001
**Egg hatching rate (%)**	95 ± 2	89 ± 2	90 ± 4	83 ± 17	-	F = 0.29; df = 3, 8; *p* = 0.835

† Numbers in the same row followed by different letters are significantly different (Tukey’s HSD (honest significant difference) test, *p* < 0.05).

**Table 3:** Effect of moxidectin on bed bug feeding, ingestion, and digestion.

Moxidectin treatment (ng/mL)		Control	20 ng/mL	40 ng/mL	ANOVA statistics
**Feeding %**	Male	98 ± 2	84 ± 13	84 ± 6	F = 0.92, df = 2, 6; *p* = 0.447
	Female	96 ± 5	93 ± 0	96 ± 2	F = 0.21, df = 2, 6; *p* = 0.820
	Nymph	97 ± 2	93 ± 2	89 ± 6	F = 1.08, df = 2, 6; *p* = 0.397
**Ingestion %**	Male	44 ± 1b †	57 ± 2a	57 ± 1a	F = 24.00; df = 2, 6; *p* = 0.001
	Female	70 ± 3	75 ± 0	73 ± 0	F = 2.79, df = 2, 6; *p* = 0.139
**Digestion % after one week**	Male	64 ± 3a	50 ± 3a	25 ± 5b	F = 25.33; df = 2, 6; *p* = 0.001
	Female	78 ± 3a	31 ± 2b	-	t = 13.81; df = 4; *p* < 0.001
**Digestion % after two weeks**	Male	95 ± 4a	95 ± 3a	70 ± 4b	F = 19.21; df = 2, 6; *p* = 0.003
	Female	-	-	-	-

† Numbers within the same row followed by different letters are significantly different (Tukey’s HSD test, *p* < 0.05).

**Table 4:** Mortalities of bed bugs after the second moxidectin feeding.

Sex/Stage	Moxidectin Treatments	*N* †	Mortality at One Week (%)	Mortality at Two Weeks (%)
**Male adult**	Control	39	0	0
	20 ng/mL	25	16	32
	40 ng/mL	10	10	10
**Female adult**	Control	28	11	14
	20 ng/mL	9	0	11
	40 ng/mL	1	0	100
**Nymph**	Control	32	3	3
	20 ng/mL	32	28	28
	40 ng/mL	6	0	0

† The number included bed bugs that were fed and survived the first moxidectin treatment, and fed during the second moxidectin treatment.

**Table 5:** Percentages of bed bug nymphs molted after the second moxidectin feeding.

Moxidectin Treatments	*N* †	Percent Molted after One Week	Percent Molted after Two Weeks
**Control**	32	87	87
**20 ng/mL**	32	0	83
**40 ng/mL**	6	50	83

† The number included bed bugs that were fed and survived the first moxidectin treatment, and fed during the second moxidectin treatment.
